# The trials and tribulations of accessing data from routine sources

**DOI:** 10.1186/1745-6215-16-S2-P225

**Published:** 2015-11-16

**Authors:** Graham Powell, Tony Marson, Dyfrig Hughes, Paula Williamson, Catrin Tudur-Smith

**Affiliations:** 1 University of Liverpool, Liverpool, UK; 2 Bangor University, Bangor, UK

## 

There are a number of administrative datasets that routinely record information on individuals in the UK. Such routine or ‘Big Data’ sources record specified data variables that are largely limited to those that enable the intended objectives of the data holder. Routine sources include clinical datasets such as Hospital Episode Statistics (HES) and the Clinical Practice Research Datalink (CPRD). In addition, non-health data is recorded by the Department of Work and Pensions (DWP), HM Revenue and Customs (HMRC) and The Driving and Vehicle Licensing Authority (DVLA) in order to meet the specified objectives of each authority.

Routine data applied to clinical research is potentially cost and resource-use effective. Clinical sources of data such as HES and CPRD are commonly accessed to provide data for retrospective observational and record-linkage studies and there are many published examples. However, there is limited evidence on the use of routine data in prospective studies, particularly randomised controlled trials (RCTs). This on-going project aims to assess the feasibility, accuracy, reliability and additional benefits of using data from routine sources for patients participating in the Standard and New Antiepileptic Drugs II RCT. Clinical and health economic outcomes will be examined from sources including HES, CPRD, DWP, HMRC and DVLA and assessed in comparison to data obtained from traditional RCT methods.

This presentation will narratively summarise the methods of access, application procedures and limitations encountered thus far with accessing individual-level, linked data from the listed routine data sources.

